# The effect of time-to-surgery on outcome in elderly patients with proximal femoral fractures

**DOI:** 10.1186/1471-2474-9-171

**Published:** 2008-12-29

**Authors:** Rüdiger Smektala, Heinz G Endres, Burkhard Dasch, Christoph Maier, Hans J Trampisch, Felix Bonnaire, Ludger Pientka

**Affiliations:** 1 University Clinic for Surgery, Department of Trauma Surgery, Knappschafts Hospital Bochum-Langendreer, Ruhr University Bochum, In der Schornau 23-25, D-44892 Bochum, Germany; 2 Department of Medical Informatics, Biometrics and Epidemiology, Ruhr University Bochum, D-44801 Bochum, Germany; 3 Department of Anesthesiology and Intensive Care, University Hospital of Münster, D-48149 Münster, Germany; 4 Clinic for Anesthesiology, Intensive, Palliative and Pain Medicine, Department of Pain Therapy, BG Klinikum Bergmannsheil, Ruhr University Bochum, Bürkle-de-la-Camp-Platz 1, D-44789 Bochum, Germany; 5 Trauma Surgery Clinic, Municipal Hospital Dresden-Friedrichsstadt, D-01067 Dresden, Germany; 6 Clinic for Geriatrics, Ruhr University Bochum, Marienhospital Herne, D-44627 Herne, Germany

## Abstract

**Background:**

Whether reducing time-to-surgery for elderly patients suffering from hip fracture results in better outcomes remains subject to controversial debates.

**Methods:**

As part of a prospective observational study conducted between January 2002 and September 2003 on hip-fracture patients from 268 acute-care hospitals all over Germany, we investigated the relationship of time-to-surgery with frequency of post-operative complications and one-year mortality in elderly patients (age ≥65) with isolated proximal femoral fracture (femoral neck fracture or pertrochanteric femoral fracture). Patients with short (≤12 h), medium (> 12 h to ≤36 h) and long (> 36 h) times-to-surgery, counting from the time of the fracture event, were compared for patient characteristics, operative procedures, post-operative complications and one-year mortality.

**Results:**

Hospital data were available for 2916 hip-fracture patients (mean age (SD) in years: 82.1 (7.4), median age: 82; 79.7% women). Comparison of groups with short (n = 802), medium (n = 1191) and long (n = 923) time-to-surgery revealed statistically significant differences in a few patient characteristics (age, American Society of Anesthesiologists ratings classification and type of admission) and in operative procedures (total hip endoprosthesis, hemi-endoprosthetic implants, other osteosynthetic procedures). However, comparison of these same groups for frequency of postoperative complications revealed only some non-significant associations with certain complications such as post-operative bleeding requiring treatment (early surgery patients) and urinary tract infections (delayed surgery patients). Both unadjusted rates of one-year all-cause mortality (between 18.1% and 20.5%), and the multivariate-adjusted hazard ratios (HR for time-to-surgery: 1.04; p = 0.55) showed no association between mortality and time-to-surgery.

**Conclusion:**

Although this study found a trend toward more frequent post-operative complications in the longest time-to-surgery group, there was no effect of time-to-surgery on mortality. Shorter time-to-surgery may be associated with somewhat lower rates of post-operative complications such as decubitus ulcers, urinary tract infections, thromboses, pneumonia and cardiovascular events, and with somewhat higher rates of others such as post-operative bleeding or implant complications.

## Background

Today, as 20 years ago, proximal femoral fractures lead to significantly reduced life expectancy and, for about 50% of patients, to an often dramatic deterioration in health and social conditions. [1-3] For many, such fractures lead to loss of independence and ultimately to institutionalization in long-term care facilities. Patients who already required some assistance prior to breaking their hip may become entirely dependent on nursing care after the fracture. [1] Are there controllable factors in the clinical care of hip fracture patients that are associated with better outcomes? The answer remains elusive.

How the timing of surgery for proximal femoral fractures affects patient outcomes has been the object of scientific discussion for years. Experienced surgeons have called for a long time to undergo surgery as quickly as possible for elderly patients with hip-fracture, on the grounds that shorter time-to-surgery is associated with reduced rates of post-operative complications and better survival rates. [2,4] In a review article published in 2003, Chilov et al. found that early surgery (within 24 to 36 hours of admission to hospital) is associated with fewer post-operative complications (pneumonia, confusion, pressure sores) and shorter hospital stays. Early surgery does not, however, lengthen patients' life expectancy. [5] In Germany, early surgical treatment of elderly patients with proximal femoral fracture is preferred, and is recommended by the guidelines of the German traumatology society.

Here we look at data from a large-scale observational study on hip fractures involving patients at 268 acute-care hospitals in Germany, to determine whether elderly patients benefit from early surgical treatment of proximal femoral fracture in terms of reduced rates of post-operative complications and improved survival prognosis.

## Methods

The present study was part of a large prospective observational study evaluating the health care situation of patients with hip and distal forearm fractures in Germany. The design and population of this study have been described previously. [6-8] Briefly, in the period between January 2002 and September 2003, data on 12,520 patients over the age of 18 admitted to rehabilitation or acute-care hospital with a femoral fracture were collected. Of these, 3,914 were treated at 268 acute-care hospitals in all regions of Germany.

The participating hospitals were spread across all regions of Germany (urban as well as rural). Therefore the patient population is broadly representative of the population of hospital patients in Germany. The size of the participating hospitals was 314 beds in median (interquartile range 197–521 beds).

Inclusion criteria for the present study were no polytrauma or coma patient, minimum age 65, isolated proximal femoral fracture (femoral neck fracture or pertrochanteric femoral fracture), first fracture event, surgical treatment in an acute-care hospital, no pathological fracture due to a malignancy, and availability of complete data for the interval between the fracture event and surgery as well as post-admission vital status. These inclusion criteria were met by 2,916 patients.

The hospital stay was documented using a standardized case report form for collecting data. Information was obtained about demographic characteristics, comorbidities, medical history, type of fracture, surgical procedure, and other aspects of medical care. The patient's overall health status was documented at the time of admission and categorized using the American Society of Anesthesiologists ratings (ASA) classification. Risk factors for osteoporosis were documented and a history of falls was taken.

Also documented were the times of the fracture event, of hospital admission, and of the start of the surgical intervention. Start of surgery within 12 hours after the fracture event was considered early surgery (short time-to-surgery), start of surgery from 12 to 36 hours after the fracture event was classified as intermediate (medium time-to-surgery), and surgery that was begun more than 36 hours after the event was considered to be late surgery (longest time-to-surgery). Details of the anesthesia and surgical procedure were documented, as were the duration of the operation and the number of blood units required.

All post-operative complications were recorded, particularly cardiovascular events, pneumonia, pulmonary embolism or thrombosis, urinary tract infections, pressure sores, post-operative bleeding requiring treatment, abscesses and implant complications (misalignment, dislocation, implant breakage, endoprosthesis luxation). One-year mortality of all causes was determined with the help of research at residency registration offices.

The recruitment and research protocols were reviewed and approved by the ethics committee of the Bavarian state medical association and the trial was undertaken in accordance with the Declaration of Helsinki. All subjects gave written, informed consent.

### Statistical analysis

All statistical analyses were performed with SAS (Version 9.1, SAS Institute Inc., Cary, NC, USA). The limit of significance was established at p = 0.05. The main predictor variable was the time between fracture event and start of surgery (time-to-surgery). The length of this interval was classified into one of three levels (I: ≤12 hours; II: > 12 to ≤36 hours; III: > 36 hours). These three levels were used as the basis for stratification of patient characteristics (Table 1), fracture type, anesthetic and surgical procedure (Table 2), post-operative complications (Table 3), and one-year all-cause mortality (Table 4). All group comparisons of categorical variables were performed using the chi-square test.

**Table 1:** Patient characteristics, stratified by time-to-surgery

Group	I	II	III	p-value (group comparison)
Time from fracture to surgery	≤ 12 h	> 12 h to ≤ 36 h	> 36 h	
Cases (total = 2916)	n = 802	n = 1191	n = 923	
Female (2324; 79.7%) n (%)	654 (81.6)	953 (80.0)	717 (77.7)	0.13

Age in years (mean, std) interquartile range	82.4 (7.5)	82.4 (7.5)	81.5 (7.2)	0.009 (I/II: n.s.; I/III: 0.04; II/III: 0.01)
	77.2–88.8	77.3–88.5	76.8–87.2	

Patients ≥ 85 years n (%)	308 (38.4)	455 (38.2)	289 (31.3)	0.001

ASA classification^A^				
ASA I/II n (%)	324 (40.4)	454 (38.3)	298 (32.3)	
ASA III n (%)	447 (55.7)	685 (57.7)	554 (60.1)	
ASA IV/V n (%)	31 (3.9)	48 (4.0)	70 (7.6)	< 0.001

Body mass index (kg/m^2^)				
< 25	462 (60.7)	669 (60.5)	544 (62.6)	
25–29.9	235 (30.9)	340 (30.8)	251 (28.9)	
≥ 30	64 (8.4)	96 (8.7)	74 (8.5)	0.88

Smoking status				
Non-smoker	676 (90.6)	981 (89.2)	759 (88.1)	
Smoker	70 (9.4)	119 (10.8)	103 (11.9)	0.25

Arterial hypertension				
No	256 (32.6)	363 (30.9)	288 (31.5)	
Yes	530 (67.4)	812 (69.1)	625 (68.5)	0.74

Diabetes mellitus				
No	594 (75.7)	918 (78.4)	674 (74.6)	
Yes	191 (24.3)	253 (21.6)	230 (25.4)	0.10

Myocardial infarction				
No	701 (93.0)	1016 (91.4)	788 (91.7)	
Yes	53 (7.0)	96 (8.6)	71 (8.3)	0.44

PAOD ^B^				
No	595 (80.7)	872 (78.4)	673 (77.4)	
Yes	142 (19.3)	240 (21.6)	196 (22.6)	0.26

Stroke				
No	640 (86.5)	949 (85.8)	716 (82.9)	
Yes	100 (13.5)	157 (14.2)	148 (17.1)	0.08

COPD ^C^				
No	658 (87.0)	976 (86.5)	780 (88.3)	
Yes	98 (13.0)	153 (13.5)	103 (11.7)	0.45

Malignancy				
No	641 (88.4)	975 (90.8)	764 (89.2)	
Yes	84 (11.6)	99 (9.2)	93 (10.8)	0.24

Creatinine > 2 mg/dl				
No	706 (91.9)	1027 (90.5)	799 (90.0)	
Yes	62 (8.1)	108 (9.5)	89 (10.0)	0.37

Type of admission				
from own home	612 (77.0)	927 (78.0)	677 (73.6)	
from nursing institution	152 (19.1)	223 (18.8)	177 (19.2)	
from clinic/hospital	31 (3.9)	38 (3.2)	66 (7.2)	< 0.001

n.s. = not significant

^A ^American Society of Anesthesiologists ratings; ^B ^peripheral arterial occlusive disease; ^C ^Chronic obstructive pulmonary disease

**Table 2:** Fracture type and operative procedure, stratified by time-to-surgery

Group	I	II	III	p-value
Time from fracture to surgery	≤ 12 h	> 12 h to ≤ 36 h	> 36 h	
Cases (total = 2916)	n = 802	n = 1191	n = 923	
Femoral neck fracture	277 (34.5)	584 (49.0)	605 (65.6)	
Pertrochanteric femoral fracture	525 (65.5)	607 (51.0)	318 (34.4)	< 0.001

TEP ^A^				
No	738 (92.0)	1039 (87.2)	789 (85.5)	< 0.001
Yes	64 (8.0)	152 (12.8)	134 (14.5)	
HEP ^B^				
No	630 (78.6)	830 (69.7)	516 (55.9)	< 0.001
Yes	172 (21.4)	361 (30.3)	407 (44.1)	
Osteosynthesis				
No	246 (30.7)	538 (45.2)	546 (59.2)	< 0.001
Yes	556 (69.3)	653 (54.8)	377 (40.8)	

Length of operation				
< 60 min	324 (41.4)	466 (40.0)	356 (39.6)	
≥ 60 to < 120 min	398 (50.9)	624 (53.6)	480 (53.5)	
≥ 120 min	60 (7.7)	74 (6.4)	62 (6.9)	0.68

Anesthesia procedure				
General anesthetic	597 (74.9)	840 (71.8)	672 (74.1)	
Regional anesthetic	200 (25.1)	330 (28.2)	235 (25.9)	0.26

Intra- and post-op. blood products				
none	377 (48.4)	532 (46.1)	444 (50.0)	
1–2 units	300 (38.5)	441 (38.2)	312 (35.1)	
3–4 units	79 (10.1)	142 (12.3)	99 (11.2)	
> 4 units	23 (3.0)	40 (3.4)	33 (3.7)	0.42

^A ^Total endoprostheses; ^B ^Hemi endoprostheses

**Table 3:** Rate of postoperative complications, requiring treatment, stratified by time-to-surgery

Group	I	II	III	p-value
Time from fracture to surgery	≤ 12 h	> 12 h – ≤ 36 h	> 36 h	
Cases (total = 2916)	n = 802	n = 1191	n = 923	
Cardiovascular complications				
No	778 (97.0)	1149 (96.5)	885 (95.9)	
Yes	24 (3.0)	42 (3.5)	38 (4.1)	0.45

Pneumonia				
No	787 (98.1)	1156 (97.1)	895 (97.0)	
Yes	15 (1.9)	35 (2.9)	28 (3.0)	0.25

Pulmonary embolism/thrombosis				
No	799 (99.6)	1185 (99.5)	914 (99.0)	
Yes	3 (0.4)	6 (0.5)	9 (1.0)	0.23

Urinary tract infection				
No	745 (92.9)	1103 (92.6)	836 (90.6)	
Yes	57 (7.1)	88 (7.4)	87 (9.4)	0.13

Bed sores				
No	797 (99.4)	1171 (98.3)	908 (98.4)	
Yes	5 (0.6)	20 (1.7)	15 (1.6)	0.10

Post-op. bleeding/Hematoma				
No	760 (94.8)	1140 (95.7)	894 (96.9)	
Yes	42 (5.2)	51 (4.3)	29 (3.1)	0.09

Abcess (epi-/subfascial)				
No	783 (97.6)	1166 (97.9)	903 (97.8)	
Yes	19 (2.4)	25 (2.1)	20 (2.2)	0.92

Implant complications				
No	777 (96.9)	1170 (98.2)	900 (97.5)	
Yes	25 (3.1)	21 (1.8)	23 (2.5)	0.14

Implant complications = misalignment, dislocation, breakage of implant, endoprothestic luxation

**Table 4:** Mortality rates, stratified by time-to-surgery

Group	I	II	III	p-value
Time from fracture to surgery	≤ 12 h	> 12 h – ≤ 36 h	> 36 h	
Cases (total = 2916)	n = 802	n = 1191	n = 923	
One-year mortality				
No	657 (81.9)	947 (79.5)	739 (80.1)	
Yes	145 (18.1)	244 (20.5)	184 (19.9)	0.40

We used Cox proportional hazards analysis for the association between the main predictor time-to-surgery and time to death from any cause within the first year after discharge. The model used was adjusted for all potential confounding factors. Potential confounding factors consisted of all medically meaningful variables independent of p-value, and of all other variables that after backward selection had a p-value of < 0.2 (Table 5). We used BMI limits below/above 22–30 kg/m^2 ^according to the European Society of Parenteral and Enteral Nutrition (ESPEN), because they are better adapted to geriatric subjects. [9] Variables are expressed as hazard ratios (HRs) with corresponding 2-sided 95% confidence intervals (95% CIs).

**Table 5:** Hazard ratios (multivariable-adjusted) for death of any causes within one year after discharge

**Model variables**	**All patients (n = 2916, death = 573)**
	HR (95% CI); p-value
Time from fracture to surgery^A^	1.04 (0.93 to 1.16); p = 0.55
Age^B^	1.05 (1.04 to 1.06); p < 0.001
Sex^C^	0.72 (0.58 to 0.88); p = 0.002
ASA^D^	1.60 (1.37 to 1.88); p < 0.001
BMI^E^	1.38 (1.16 to 1.65); p < 0.001
Malignancy^F^	1.84 (1.46 to 2.32); p < 0.001
Kidney dysfunction^G^	1.34 (1.05 to 1.71); p = 0.02
COPD	1.39 (1.11 to 1.75); p = 0.004
Post-op complication – cardiovascular	1.87 (1.33 to 2.63); p < 0.001
Post-op complication – decubitus ulcers	2.08 (1.20 to 3.58); p = 0.009
Post-op complication – stroke	2.37 (1.05 to 5.32); p = 0.037
Post-op complication – implant	0.97 (0.54 to 1.75); p = 0.92

^A ^0 ≤ 12 h, 12 h < 1 ≤ 36 h, 2 > 36 h; ^B ^age in years; ^C ^1 = men 2 = women; ^D ^three ASA groups (American Society of Anesthesiologists ratings): 1 = ASA1 or ASA2, 2 = ASA3, 3 = ASA4 or ASA5; ^E ^Body mass index according to ESPEN limits for age 65+ (0: 22.0 to < 30.0; 1: < 22.0 or ≥ 30.0); ^F ^malignancy in patient history; ^G ^kidney impairment in patient history;

A possible association between post-operative complications and time-to-surgery was tested using a multiple logistic regression analysis. Adjusted measures of risk (odds ratios with corresponding 2-sided 95-percent confidence intervals) were calculated (Table 6).

**Table 6:** Risk of post-operative complications (multivariable-adjusted) as a function of time-to-surgery

**Post-operative complication**	**Time from fracture to surgery^A^** **OR (95% CI); p-value**
Pulmonary embolism/thrombosis	1.72 (0.85–3.50); p = 0.133
Cardiovascular	1.13 (0.87–1.48); p = 0.369
Pneumonia	1.16 (0.85–1.58); p = 0.365
Implant	0.91 (0.66–1.26); p = 0.562
Vascular injury	0.24 (0.03–1.80); p = 0.165
Bleeding	0.75 (0.59–0.97); p = 0.026
Bed sores	1.33 (0.86–2.05); p = 0.201

^A ^Time-to-surgery interval I (≤ 12 h) vs. II (> 12 h–≤ 36 h) vs. III (> 36 h); OR = odds ratio; CI = confidence interval, multivariable-adjusted for age, sex, ASA categories, BMI ESPEN, diabetes, and hypertension

## Results

### Patient characteristics

Patient characteristics, stratified by time-to-surgery, are summarized in Table 1. Of the 2916 hip-fracture patients, 802 (27.5%) were operated on within 12 hours of the fracture event, 1191 (40.8%) in 12 to 36 hours, and 923 (31.7%) more than 36 hours after the fracture event. The mean age (SD) of patients was 82.1 (7.4) years. The proportion of patients over 85 years in the late surgery group was somewhat smaller (31.3%) than in the two shorter time-to-surgery groups (38.4% and 38.2%). Women made up 79.7% of the study population. At the time of hospital admission, the attending physician documented the patient's overall health status. Over half of study participants were classified as ASA III. The percentage of multimorbid patients (ASA classification IV/V) was somewhat higher in the late surgery group than in the other two groups (7.6% vs. 3.9% and 4.0%).

### Fracture type and operation

Among patients in the short or medium time-to-surgery groups, pertrochanteric femoral fracture was the most common type of injury (65.5% and 51.0%) (Table 2). By contrast, femoral neck fractures were considerably more frequent in the group with the longest time-to-surgery (65.6%). Osteosynthetic procedures were more frequent in the shorter time-to-surgery group than in those for whom surgery was delayed (69.3% vs. 54.8% vs. 40.8%; p < 0.001). With respect to endoprosthetic procedures, hemi-endoprostheses were implanted about three times more often than total endoprostheses. Both endoprosthetic procedures are seen significantly more frequently in the late surgery group.

At 72.3% of all cases, general anesthetic was preferred over regional anesthetic procedures. In slightly less than half of all operations, no blood products were needed either intra- or postoperatively. Practically all pertrochanteric fractures were treated by osteosynthetic procedures. In more than half of patients with pertrochanteric femoral fracture the operation lasted less than an hour, whereas for patients with femoral neck fracture the procedure generally took between 60 and 120 minutes (data not shown). In around 93% of patients the operation lasted less than 2 hours, independent of time-to-surgery.

### Post-operative complications

Table 3 shows the observed frequencies of post-operative complications, stratified by time-to-surgery. Complications were recorded by doctors only if they required treatment. The complications are either general in nature (e.g. cardiovascular complications) or specific to the operation performed (e.g. post-operative bleeding). At 232 cases (8.0%), urinary tract infections were the single most frequent post-operative complication. Post-operative bleeding requiring treatment occurred in 122 cases (4.2%) and cardiovascular complications in 104 (3.6%). Seventy-eight patients (2.7%) developed pneumonia after surgery, implant-related complications occurred in 69 cases (2.4%) and 64 patients (2.2%) developed abscesses. Pressure sores (1.4%) and thrombosis/pulmonary embolism (0.6%) were rare overall. Group comparison showed no significant differences in frequencies of the various post-operative complications. Patients who received early surgery were somewhat more likely to develop post-operative bleeding or implant complications, while those in the longer time-to-surgery group were somewhat more likely to suffer from pressure sores, urinary tract infections, thromboses or pneumonia.

### Mortality

One year after discharge from hospital, 573 patients (19.7%) had died. Statistically significant differences in mortality as a function of time-to-surgery were not observed (Table 4).

### Survival analysis

Mortality risk (hazard ratio) as a function of time-to-surgery was calculated by means of Cox regression. Table 5 shows the result after multivariate adjustment. The factors with the greatest influence on mortality risk are, after age (HR = 1.05 per year of age), the aforementioned postoperative complications (cardiovascular, pressure sores, stroke), all of which are indicative of poor cardiovascular health in general, as well as a prior history of cancer and poorer ASA classification.

Female sex is a protective factor (HR = 0.72), and implant complications have no influence (HR ≈ 1.0) on mortality after hospital discharge.

In Table 6, multivariate logistic regression was used to analyse associations between time-to-surgery and post-operative complications. Longer time-to-surgery was a protective factor only in relation to post-operative bleeding (OR = 0.75; p = 0.026). For all other post-operative complications, delayed surgery was not associated with any statistically signficant increase in risk.

## Discussion

Hip fracture represents a major prognostic risk factor for elderly people. Research shows a one-year mortality of these patients of 20–25%. It is also known that after a fracture event, elderly patients do not regain their previous level of activity, which can have effects up to a complete loss of social independence. [1,5,10,11] According to studies by Roberts and Goldcare, excess mortality in hip fracture patients has remained almost unchanged in 20 years. [12] Besides age, negative prognostic factors are considered to be male sex, poor overall health status (high ASA classification), cognitive impairment, and a low Barthel index. [13] The question of whether early surgical treatment of elderly patients with hip fracture has a significant effect on the patient's prognosis is still subject to scientific debate.

The issue has been addressed in numerous scientific publications. [13-18] In their guidelines project published in 1999, March et al. found that the overall length of hospital stay and risk of postoperative complications both increase with the length of time from hospital admission to the start of surgery. [17] In stable patients, proceeding rapidly to surgery is not associated with any additional risk. Our own prospective study of 1393 patients confirmed this finding. [19]

In a prospective observational study of 2,660 consecutive patients, Moran found that patients with relevant comorbidities who received delayed surgery had a 2.5 higher mortality risk than patients without comorbidities who received the same treatment. For patients without comorbidities, they found that mortality increases only if surgery was delayed beyond the fourth day in hospital. [20] The statistical significance of these results is limited, however, since only 28 patients had surgery that was delayed beyond the fourth day. The prospective study pointed to other outcome-relevant factors: patients who suffered from both acute pneumonia and hip fracture had a significantly increased mortality, during their stay in hospital as well as in the first year post-fracture. Patients identified as "stable" were operated on quickly. The authors concluded, however, that the identification of patients presenting with hip fracture as "stable" or "unstable" is made at the discretion of the individual physician, and even in their study was not done according to standardized criteria.

In a prospective observational study of 367 patients, Zuckerman identified surgery after the second day following admission to hospital as a risk for patients. The central finding was that mortality within the first year after fracture doubled for elderly patients (who are cognitively intact, and living at his or her own home prior to hip fracture) if surgery was delayed for three calendar days or more from the time of admission to the time of surgery. [4]

In an observational study of 18,209 patients McGuire also found that a delay between admission and surgery of two days or more was associated with significantly increased mortality, and Gdalvich also confirms this. [21,22] In his prospective observational study, Moran found a significant increase in mortality when surgery was performed on the fourth day of the hospital stay. [20] In another prospective observational study, Doruk identified the fifth day following admission as being prognostically relevant. [23]

Other researchers have further differentiated the time-to-surgery. Based on the results of a prospective observational study, Dorotka call for hip-fracture patients to be operated on within 6 hours of admission to hospital. Patients who received surgery within the first 6 hours after admission had a mortality of 10.1% after six months. If surgery was delayed beyond 6 hours, the six-month mortality rose to 21.8%. The difference was statistically significant. The two groups of patients (< 6 hours, > 6 hours) did not differ significantly with regard to age and comorbidities. [24] Kenzora on the other hand identifies surgery within the first 24 hours as a risk factor for patients: he found that if surgery was performed within 24 hours, one-year mortality was 34%, whereas if it was delayed to between the second and fifth day, it ranged from 6 to 11%. [25]

Orosz reports on a prospective cohort study of 1206 patients that found no effect of time-to-surgery on survival, but surgery within 24 hours was associated with shorter hospital stays, less patient pain and lower rates of post-operative complications. It should be noted, however, that only "stable" patients received early surgery, and that the definition of "stable" was up to the individual physician. Also, functional status was not better in patients who received surgery within 24 hours than it was in those for whom surgery was delayed. [15]

In our study we show that in elderly patients (≥65 years) who have suffered an isolated proximal femoral fracture, short time-to-surgery (within 12 hours after the fracture event) does not result in better survival prognosis compared with longer (12–36 hours) or very long (more than 36 hours) time-to-surgery. For our comparison, we have deliberately recorded the time from the fracture event to the operation, in order to take into account those cases in which a patient who is living alone is only discovered late after the fracture event. It is to be expected that the outcome for these patients is poorer, even if very little time elapses between admission to hospital and time of operation.

Although a group comparison showed that certain post-operative complications are more frequent in the longest time-to-surgery group (whereas others such as post-operative bleeding tended to somewhat more frequent in the shortest time-to-surgery group), these differences in the rates of complications were not statistically significant. It is possible that statistically significant differences in these frequencies would have been found in a larger patient population. Nevertheless the frequency differences for serious complications (cardiovascular events, pneumonia, embolisms) between shortest and longest times-to-surgery were very small, at around 1%.

## Conclusion

The literature analysis shows that studies looking at the effect of time-to-surgery on outcomes for patients suffering from hip fracture have produced inhomogeneous findings. Our own study with 2916 representative unselected hip-fracture patients aged 65 years or older shows a trend toward more frequent post-operative complications in the longest time-to-surgery group, but no effect of time-to-surgery on mortality.

A subject that merits further research is that of the so-called "stable" patients, a specific subgroup of hip-fracture patients that, in the view of numerous authors, benefits from the shortest possible time-to-surgery.

## Competing interests

The authors declare that they have no competing interests.

## Authors' contributions

RS conceived of the study, participated in the design of the study, in interpretation of the data and drafting of the manuscript. HGE performed the statistical analysis, participated in the design of the study, in interpretation of the data and drafted the manuscript. BD participated in the statistical analysis and in critical revision of the manuscript. CM participated in the design of the study, in interpretation of the data and drafting of the manuscript. HJM participated in the design of the study, in obtaining funding, in the statistical analysis and in critical revision of the manuscript. FB participated in interpretation of the data and drafting of the manuscript. LP participated in the design of the study, in obtaining funding, in interpretation of the data and drafting of the manuscript. All authors read and approved the final manuscript.

## Pre-publication history

The pre-publication history for this paper can be accessed here:


